# Air exposure moderates ocean acidification effects during embryonic development of intertidally spawning fish

**DOI:** 10.1038/s41598-022-16399-6

**Published:** 2022-07-18

**Authors:** Andrea Y. Frommel, Sadie L. R. Lye, Colin J. Brauner, Brian P. V. Hunt

**Affiliations:** 1 Faculty of Land and Food Systems, University of British Columbia, 2357 Main Mall, Vancouver, BC V6T 1Z4 Canada; 2 Institute for the Oceans and Fisheries, University of British Columbia, 2202 Main Mall, Vancouver, BC V6T 1Z4 Canada; 3 Department of Zoology, University of British Columbia, 6270 University Boulevard, Vancouver, BC V6T 1Z4 Canada; 4 Department of Earth, Ocean and Atmospheric Sciences, University of British Columbia, 2207 Main Mall, Vancouver, BC V6T 1Z4 Canada; 5 Hakai Institute, Quadra Island, PO Box 309, Heriot Bay, BC V0P 1H0 Canada

**Keywords:** Ecology, Zoology, Climate sciences, Ecology, Environmental sciences, Ocean sciences

## Abstract

Ocean acidification can negatively impact the early life-stages of marine fish, due to energetic costs incurred by the maintenance of acid–base homeostasis, leaving less energy available for growth and development. The embryos of intertidally spawning fishes, such as Pacific herring, are often air exposed for hours. We hypothesized that air exposure would be beneficial to the developing embryo due to a higher oxygen availability (and thus reduced metabolic costs to secure adequate oxygen) and permitting excess CO_2_ associated with ocean acidification to be off-gassed during emersion. To investigate this, we reared Pacific herring (*Clupea pallasii*) embryos under three tidal regimes (subtidal: fully immersed, low intertidal: 2 × 2 h air exposure, and high intertidal: 5 + 9 h air exposure) fully crossed with three aquatic CO_2_ levels (400, 1500 and 3200 µatm) at a water temperature of 9.5 °C and naturally fluctuating air temperature during air exposure. We measured the effects on embryonic development and hatch, as well as carry-over effects on larval development and survival. Air exposure during embryonic development had significant positive effects on growth, condition and survival in larval Pacific herring, with some interactive effects with CO_2_. Interestingly, CO_2_ by itself in the fully immersed treatment had no effect, but had significant interactions with air exposure. Our research suggests that air exposure during low tide can be highly beneficial to intertidally spawning fishes and needs to be taken into account in climate change studies and modeling.

## Introduction

The eastern North Pacific is an ocean region particularly impacted by climate drivers, where high CO_2_ levels shoaling to the surface with upwelling are an important feature of the ocean dynamics^1–3^. Pacific herring (*Clupea pallasii*) are a keystone species in the North Pacific as an important forage fish in the marine food web^4^. Being one of the main prey items of Pacific salmon, which in turn are the main prey of resident orcas (*Orcinus orca*), they play a key role in the ecosystem, economy and culture in the Pacific Northwest^4^. Pacific herring stocks have shown substantial fluctuations in the last two decades, and some management units are at all time low levels, with direct and indirect effects of climate change likely contributing to their decline^5–7^. Year-class strength is determined by survival of the vulnerable larval stage, which in turn is strongly dependent on environmental factors affecting successful development^8^. One such environmental factor is ocean acidification (OA), to which the NE Pacific may be particularly vulnerable due to naturally high concentrations of aquatic CO_2_ from both natural and anthropogenic drivers including upwelling, low buffering capacity, riverine input and eutrophication^9–14^. Detrimental effects of future projections of ocean acidification levels have been documented in experiments with embryonic and larval Pacific and Atlantic herring (*Clupea harengus*), with lowered condition factor, developmental delays and malformations reported at CO_2_ levels as low as 1000 µatm^15–18^, levels which already occur episodically in coastal regions including the Salish Sea^19–22^. These effects are likely a result of increased energetic costs associated with maintaining acid–base homeostasis in elevated environmental CO_2_^23,24^.

However, Pacific herring are intertidally spawning fishes and as a result, the embryos are periodically exposed to air during low-tide, in which gas exchange of oxygen and CO_2_ between the embryo and the atmosphere occurs. While the physiological effects of tidally fluctuating CO_2_ levels have been studied to some extent in larval fish^25^, the effects of air exposure have not been included. We propose that air exposure during embryogenesis may play an important role in counteracting negative effects of high marine CO_2_ levels in the wild.

Pacific herring spawn in winter in the intertidal and upper sub-tidal zones, synchronized with the tides, on marine vegetation in sheltered regions and estuaries. Spawning height is dependent on tidal height and beach slope^26^, and also female choice with larger females tending to deposit larger eggs higher in the intertidal as a form of parental care where the embryos are exposed to air for several hours per day^27^. Tide pools and estuaries can become hypoxic at low tide, particularly at night, and thus fishes that spawn in air exposed regions during low tide avoid hypoxic conditions for their embryos. The greater oxygen availability and diffusivity in air can be further advantageous to embryos, by accelerating development at reduced metabolic costs^28^. Air-reared embryos of the mangrove killifish, *Kryptolebias marmoratus,* consumed nearly half the amount of oxygen and had larger yolk reserves compared to water-reared embryos while maintaining comparable growth rates, indicating highly reduced energetic costs of development in air^29^. In addition to a higher oxygen availability, air exposure may allow the embryos to off-gas excess CO_2_, thus temporarily alleviating some of the negative effects of ocean acidification. While air exposure has been found to increase mortality in Pacific herring^27^ and capelin (*Mallotus villosus*)^30^, this has mainly been ascribed to desiccation and temperature stress. For example, in Pacific herring, air exposure had no effect on survival in embryos at 5 and 8 °C, with a mean 80% survival, while 5–8 h daily air exposure at 11 °C decreased survival to as low as 5%^31^. For Pacific herring spawning on the coast of British Columbia (BC) in winter, high temperatures and desiccation may not be a common problem given the seasonal cold temperatures (typically 0–10 °C) and high humidity (typically 40–100%) that they experience at that time of year^32^.

**Figure 1:**
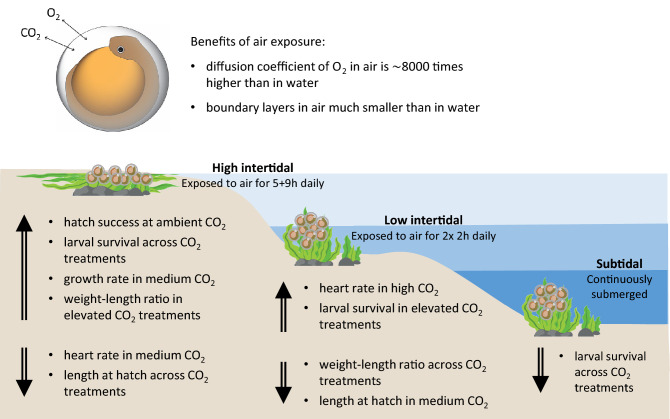
Graphical abstract summarizing the findings on the combined effects of intertidal air exposure and CO_2_.

In this study, we hypothesized that air exposure during embryonic development of Pacific herring would reduce negative effects of ocean acidification on embryonic development and hatch success, with carry-over effects on larval growth, development and survival. To address this, fertilized herring eggs were collected from the wild and brought into the lab where they were exposed to one of three CO_2_ treatments (1) 400 µatm, (2) 1600 µatm and (3) 3000 µatm, designed to mimic control conditions, medium CO_2_ conditions present during winter mixing and high CO_2_ levels predicted with climate change, respectively. These three CO_2_ treatments were fully crossed with one of three air exposure treatments during embryonic development (1) subtidal: fully immersed, (2) low intertidal: 2 × 2 h day^−1^ and (3) high intertidal: 5 h + 9 h day^−1^. For ecological relevance, all air exposure treatments were conducted in naturally fluctuating air temperature and ambient humidity. We hypothesized that (H1) CO_2_ would negatively affect embryonic and larval growth, survival and development, cause cranial and spinal malformations and increase heart rate; (H2) Moderate air exposure in the low intertidal (2 × 2 h air exposure day^−1^) would be beneficial to the embryos improving growth and survival and counteract the negative effects of elevated CO_2_ described in H1; (H3) Prolonged air exposure in the high intertidal (5 h + 9 h air exposure day^−1^) would counteract negative effects of elevated CO_2_ on embryonic development and expected cranial and spinal malformations, but result in synergistic negative effects on larval growth and survival. A graphical abstract is shown in Fig. 1.

## Material and methods

### Egg collection and husbandry

Wild spawned herring eggs attached to algae were collected from Williams Beach, Vancouver Island, British Columbia on March 5, 2021 at 1 pm during the receding tide. Eggs were brought back to the Hakai Marine Station, Quadra Island, British Columbia in coolers where they were sorted for viability and staged to be between e and f, approximately 2.5 days post fertilization (dpf)^33^, with 90% epiboly but no optic vesicle or myomeres apparent. Live embryos were randomly distributed into 27 mesh hatch boxes (Pen Plax NB2 Net Breeder Deluxe; dimensions: 2.79 × 14.22 × 28.96 cm) at a density of approximately 300 eggs per hatch box. These were in turn floated in one of 9 tanks (3 hatch boxes per tank) randomly set to three different CO_2_ treatments in triplicate: (1) control CO_2_ at 400µatm, (2) medium CO_2_ at 1600 µatm and (3) high CO_2_ at 3000 µatm. The treatment tanks were 380 L glass aquaria with a 260 L holding tank in front and a 120 L back chamber which was used for chilling and CO_2_ equilibration. Tanks were set to partial flow-through with filtered seawater taken from 20 m depth in Hyacinth Bay, and partial recirculation with water from the back chamber. Target CO_2_ tensions were accomplished by equilibrating water with fine air diffusers with a pre-set CO_2_-air mixture using mass-flow controllers in the back chamber and circulated through the holding tank. Each tank had a individual pH and temperature meters recording values every 5 min. Water temperature was maintained at 9.5 °C with an industrial chiller coil in the back chamber, which matched the ocean temperature at which the embryos were collected. Oxygen, salinity, temperature and pH were monitored daily with handheld meters (YSI), and ammonia was monitored every other day and remained below 0.1 ppm.

Each of the 3 hatch boxes floating in each replicate tank was assigned to one of three air exposure regimes: (1) continuous immersion, (2) 2 h day and 2 h night air exposure (2 × 2 h day^−1^) and (3) 5 h day and 9 h night air exposure (5 h + 9 h day^−1^), corresponding to what herring embryos would experience in the (1) subtidal, (2) low intertidal and (3) high intertidal, resulting in a fully crossed 3 air-exposure × 3 CO_2_ treatments in triplicate (27 hatch boxes). During air-exposure, hatch boxes were placed into a communal shaded, dry sea table outside where they were exposed to natural temperature fluctuations throughout the day and night. Air temperature was monitored throughout. Each day, dead embryos were counted and boxes were checked for hatched larvae.

At first signs of hatch, embryos were transferred from the hatch boxes into 2 L glass jars covered with fine mesh and immersed back into the same 9 aquaria for CO_2_ equilibration (27 glass jars in total), in order to prevent escape, keeping each treatment and replicate separate. One day post peak hatch, algae were removed from the jars and all egg shells, dead and unhatched embryos were counted and removed. Each day, dead larvae were removed via siphon and counted. Every other day, pH, temperature, salinity, oxygen and ammonia were measured in all individual jars and 25% of the water in each jar was exchanged with the corresponding tank water. Discreet water samples were taken for carbonate chemistry measurements on day 1, 6 and 15 of the experiment.

At 5 days post hatch (dph), a mass mortality occurred in one jar of the control CO_2_, high intertidal (5 + 9 h air exposure) treatment, where 89% of the larvae died for reasons unknown. Oxygen, ammonia and pH levels were normal and water appeared clear. As the other two replicates were unaffected, larvae from this jar were removed from further analysis.

### Sampling and analysis

**Figure 2:**
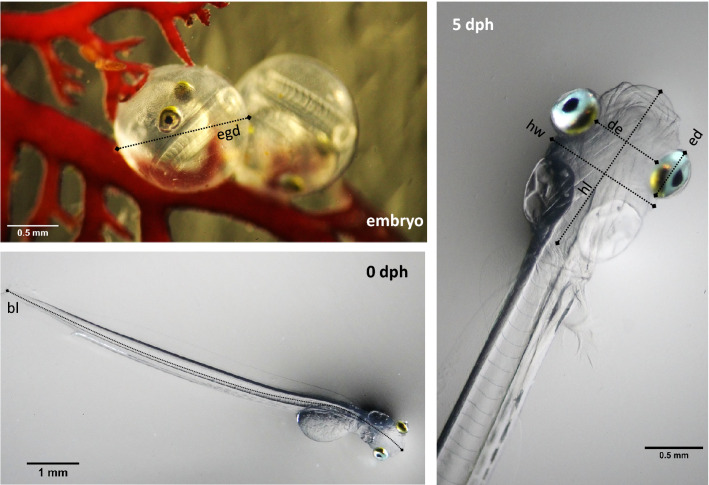
Morphology and measurements of embryonic and larval herring. Embryonic and larval herring: embryo at 6 days post fertilization (dpf; upper left panel) used for heartbeat frequency and egg diameter measurements, larvae at hatch (0 days post-hatch (dph); lower left panel) and 5 dph (right panel), used for length measurements, morphometrics and screening for cranial abnormalities (*egd* egg diameter, *bl* body length, *hw* head width, *hl* head length, *de* distance between eyes, *ed* eye diameter).

Herring embryos were sampled at 6 dpf which corresponded to 4 days after transfer into treatment. Four embryos per hatch box were photographed live in a dish of water from their tanks under a dissecting microscope (Zeiss Stemi 508 with a mounted Sony A7R4) at 0.8× and 1.6× magnification for staging and morphometrics. Heart beats were video taped at 1.6× magnification for 10 s for calculation of heart rate as an indicator of metabolic rate. Water temperatures in the dishes were monitored and were consistently 12 °C. Embryos were then transferred to Eppendorf caps, frozen in liquid nitrogen and stored at − 80 °C for further analysis. Larvae were sampled at hatch (0 dph), 3 dph and 5 dph. Ten larvae per jar were photographed live under the microscope at 0.63× and 1.6× magnification for morphometrics and length measurements, transferred individually into caps, frozen in liquid nitrogen and stored at − 80 °C for further analysis. The experiment was terminated at yolk-sac depletion (6 dph) at which point all larvae were counted out and a subsample frozen at − 80 °C. Frozen herring larvae were weighed, freeze-dried overnight and weighed dry the next day on a high precision microbalance (Satoris). From the images, larval length, body depth, body area, yolk sac area, eye diameter, distance between eyes and the head length and width were measured as indicators for development (Fig. 2) using image J. Growth rates (of body area, length and weight) and yolk sac depletion rates (from yolk sac area measurements) were calculated as a measure of energy transfer of yolk into growth. Weight and length measurements were used to calculate Fulton’s condition factor K. Larval developmental stage was determined after Kawakami et al.^33^ for each larvae and larval images were assessed for cranial and spinal malformations using symmetry and head dimensions for cranial assessment and straightness for spinal assessment.

All analyses were done blind to the treatment from which they came and in random order.

### Animal care

This study was carried out under strict accordance with the animal care protocol approved by the University of British Columbia Animal Care Committee (certificate # A19-0284). Wild embryos were collected under the Fisheries and Oceans permit number XR 57 2021.

### Statistics

All statistics were done in R^34^ and plots were generated using ggplot. After testing for normality and homogeneity of variance, main effects and interactions of CO_2_ and tidal exposure were tested with linear mixed models, including hatch box or jar—henceforth known as “replicate”—as a random factor, using the lmer function from the package lme4, with the outputs of p-values and Chi square (*X*^2^). Contrasts in tidal regime across fixed CO_2_ levels were made using packages phia and multcomp. P-value adjustments were made using the holm method. Percent hatched and dead larvae were arcsin-transformed and percent daily embryonic mortality was ln-transformed for normal distribution and analyzed using a multifactorial ANOVA, giving p- and F-values. Effect size analyses were performed by calculating the natural logarithm of the response ratio (LnRR) of the air exposure treatments relative to the subtidal regime with 95% confidence intervals at each of the three CO_2_ levels^35^. Effects of treatment on correlation of morphometrics (body length, head width, head length, distance between eyes, eye diameter and cranial deformities) were tested with a principal component analysis using the packages devtools and factoextra.

## Results

### Abiotic parameters

**Figure 3:**
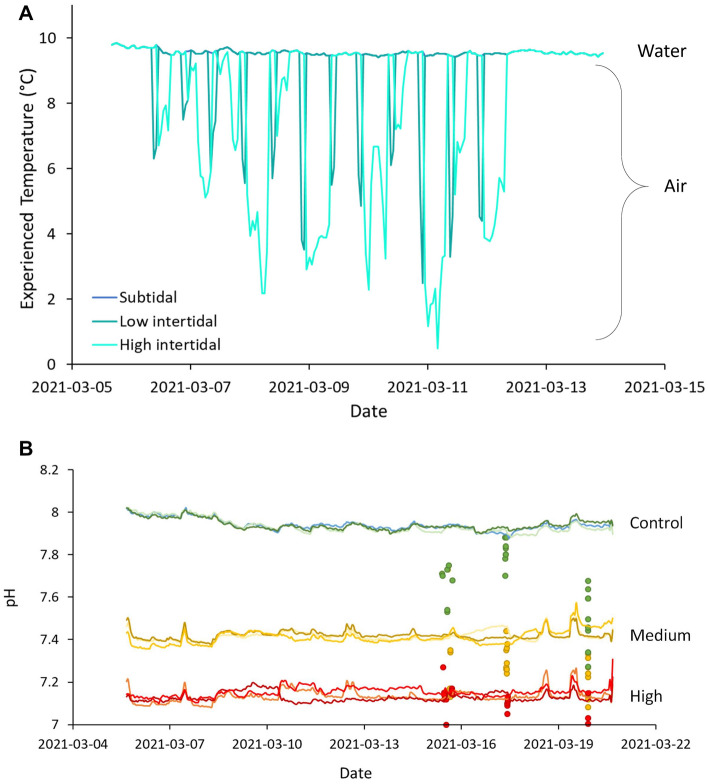
Temperature and pH experienced by the herring embryos and larvae throughout the experiment. Hourly measurements of (**A**) air/water temperature experienced by herring embryos in each of the tidal treatments (subtidal: continuous immersion in 9.5 °C water; low intertidal: 2 × 2 h air exposure; high intertidal: 5 + 9 h air exposure) and (**B**) pH levels in the tanks for each of the CO_2_ treatments (greens = control, 400 µatm CO_2_, yellows = medium, 1600 µatm CO_2_, reds = high, 3000 µatm CO_2_); dots are pH levels measured in the individual jars during larval incubation.

The temperature regime experienced by the embryos was purposefully natural and therefore varied between the three air exposure treatments. The subtidal treatment, where embryos were continuously submerged in water, remained around 9.5 °C for the duration of the experiment, while the intertidal treatments experienced dips in temperature during outside air exposure down to 2.5 °C and 0.8 °C, for the low and high intertidal respectively (Fig. 3A). Accumulated thermal units (ATU; days × temperature post collection until hatch) for each air exposure treatment were 79.6, 75.4 and 65.3 for subtidal, low intertidal and high intertidal, respectively. Despite differences in thermal regime, peak hatch was on the same day (March 14, 2021) for all air exposure and CO_2_ treatments, estimated at 11 dpf.

The pH levels in the tanks were measured hourly and were stable over the course of the embryonic incubation period, with no overlap between treatments, although there was some overlap between individual jars. Control treatment was consistently around a pH of 8, the medium treatment had a pH of 7.4 and the high CO_2_ treatment had a mean pH of 7.1 (Table 1). After hatch, when the larvae were transferred to the jars, circulation and gas exchange between jars and tank were not as high and CO_2_ accumulated in the jars over time, leading to pH levels deviating from tank pH levels (Fig. 3B). Although oxygen levels remained high (7–9 mg/L), the pH dropped from a mean 8–7.6 in the control on two occasions, and was brought back up with a partial water exchange from the incubation tank water. The pH in the medium and high CO_2_ treatments were not as affected (Fig. 3B), however, final water chemistry measurements after completion of the experiment (2 days post water exchange) revealed much higher CO_2_ levels in all treatments (Table 1: day 15).

**Table 1:** Mean water parameters for each treatment (mean of 3 tanks ± S.D.) at the beginning (day 1, 2021-03-06) and end (day 6, 2021-03-12) of embryonic incubation and mean parameters in the jars (N = 9) at the end of larval incubation (day 15, 2021-03-19); Temperature (T), salinity, *p*CO_2_, total CO_2_ (TCO_2_) measured at distinct sampling intervals with the BoL; total alkalinity (TA) and pH (on the total scale) calculated with CO2SYS.

Day	CO_2_ treatment	T (°C)	Salinity	*p*CO_2_ (µatm)	TCO_2_ (µmol/kg)	TA (µmol/kg)	pH_Total_
1	Control	9.73 ± 0.23	28.63 ± 0.06	386.74 ± 11.93	1883.02 ± 6.94	2010.11 ± 4.53	8.02 ± 0.01
1	Medium	9.80 ± 0.26	28.70 ± 0.00	1658.61 ± 37.65	2075.11 ± 3.05	2041.48 ± 2.18	7.44 ± 0.01
1	High	9.77 ± 0.25	28.67 ± 0.06	3290.81 ± 150.21	2170.92 ± 14.87	2042.42 ± 6.32	7.15 ± 0.02
6	Control	9.63 ± 0.29	28.80 ± 0.00	442.21 ± 6.06	1912.95 ± 6.23	2024.27 ± 3.48	7.97 ± 0.01
6	Medium	9.53 ± 0.06	28.80 ± 0.00	1628.72 ± 4.93	2068.54 ± 1.81	2035.82 ± 2.07	7.44 ± 0.00
6	High	9.60 ± 0.17	28.80 ± 0.00	3094.61 ± 61.82	2166.03 ± 3.54	2047.18 ± 5.6	7.17 ± 0.01
15	Control	9.70 ± 0.09	28.70 ± 0.05	1500.41 ± 523.64	2066.71 ± 52.4	2048.15 ± 17.62	7.50 ± 0.14
15	Medium	9.44 ± 0.14	28.74 ± 0.07	2927.64 ± 642.29	2159.41 ± 55.56	2049.57 ± 23.17	7.21 ± 0.09
15	High	9.61 ± 0.23	28.76 ± 0.09	5344.02 ± 1054.55	2306.01 ± 65.14	2075.08 ± 16.83	6.95 ± 0.09

### Effect of air exposure and CO_2_ treatment during embryonic development

**Figure 4:**
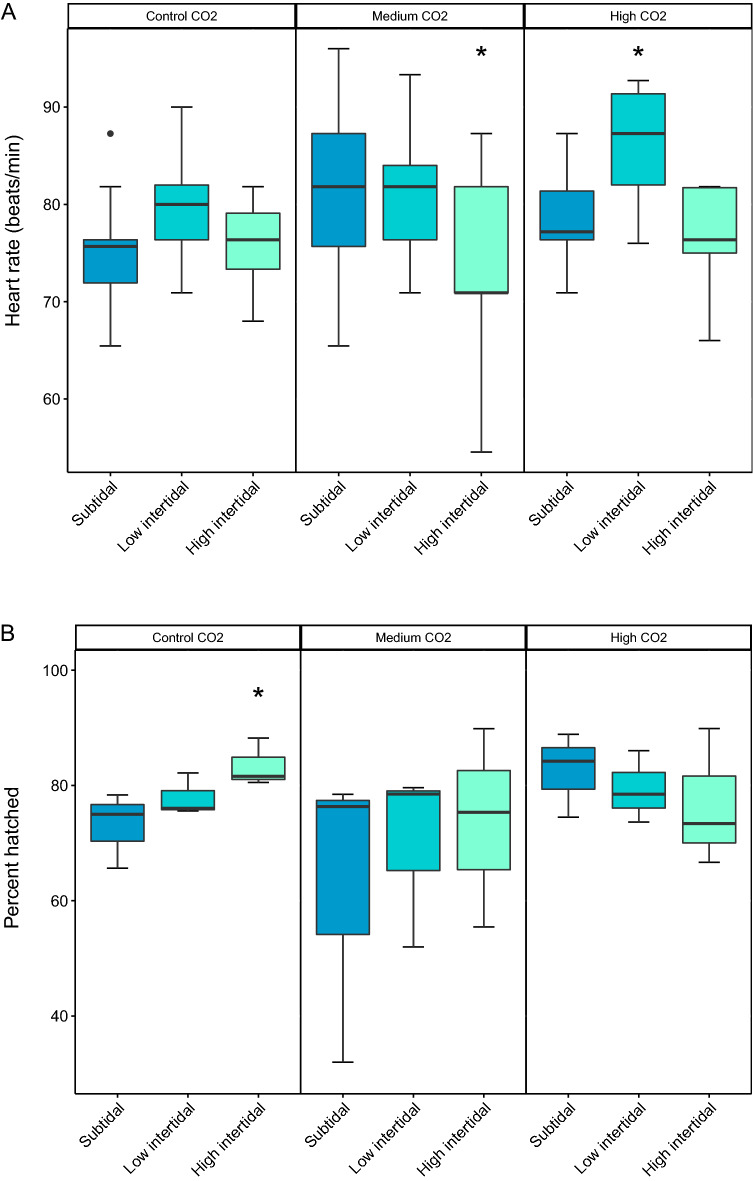

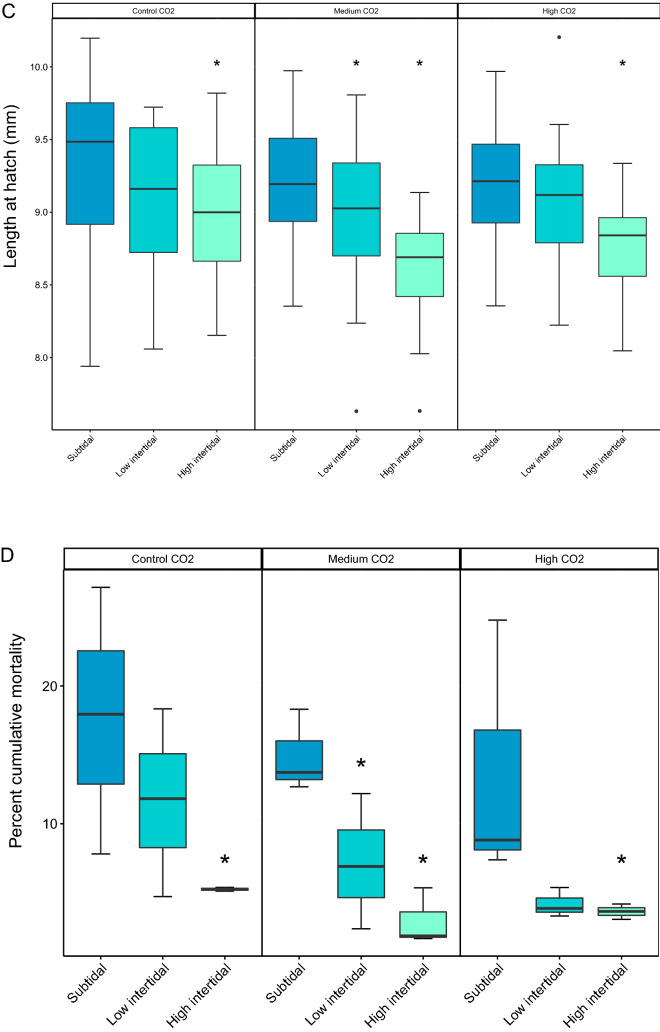
Embryonic heart rate, hatch success, length at hatch and cumulative mortality show a complex response to CO_2_ and air exposure. Box-whisker plots showing the 25th and 75th percentile (box), the median (line), the 0 and 100th percentile (whiskers) and outliers (points) of (**A**) Embryonic heart rate, (**B**) hatch success, (**C**) length at hatch and (**D**) percent cumulative mortality to 6 dph; for three tidal regimes (subtidal = no air exposure; low intertidal = 2 × 2 h air exposure day^−1^; high intertidal = 5 + 9 h air exposure day^−1^) at three different aquatic CO_2_ rearing conditions (Control = 400µatm; Medium = 1600µatm; High = 3000 µatm CO_2_). Stars above box-whisker plots denote significant differences relative to the subtidal (fully immersed treatment) at the respective CO_2_ level (effect size statistics in Table 3).

Neither embryonic survival nor growth were significantly affected by treatment in our experiment. Percent daily embryonic mortality was low and not significantly affected by CO_2_ treatment or air exposure (CO_2_: p = 0.088, F_2_ = 2.45; Tide: p = 0.11, F_2_ = 2.19; CO_2_*Tide: p = 0.18, F_2_ = 1.59) . Egg diameter at 6 dpf was also not significantly affected by treatment (CO_2_: p = 0.38, *X*^2^ (2, N = 30) = 1.92; Tide: p = 0.83, *X*^2^ (2, N = 30) = 0.33; CO_2_*Tide: p = 0.08, *X*^2^ (2, N = 30) = 8.25). Metabolic rate, as indicated by embryonic heart rate, was significantly affected by air exposure at 6 dpf (p < 0.001, *X*^2^ (2, N = 12) = 17.38), but not CO_2_ (p = 0.09, *X*^2^ (2, N = 12) = 4.74 or CO_2_*Tide (p = 0.15, *X*^2^ (2, N = 12) = 6.66. Interestingly, in the medium CO_2_ treatment, embryonic heart rate was significantly lower in the high intertidal treatment compared to the low intertidal and subtidal treatment (p = 0.027, *X*^2^ (2, N = 12) = 8.6), while in the high CO_2_ treatment, heart rate was significantly increased by 10% in the low intertidal compared to the subtidal and high intertidal regime (p = 0.007, *X*^2^ (2, N = 12) = 12.0) (Fig. 4A, Tables 2 and 3).

**Table 2:** Linear mixed model including replicate (hatchbox/jar) as a random factor, showing the main effect of CO_2_ across intertidal treatments relative to the subtidal. Below are the summary of Chi square tests (*X*^2^) and p-value using the adjustment method Holm. N refers to number of embryos/larvae per treatment summed across replicates.

Parameter	CO_2_	Low intertidal	High intertidal	Df	N	*X*^2^	*p*
Heart rate	Control	− 0.61	4.05	2	12	3.44	0.179
	Medium	7.65	7.94	2	12	8.60	0.027*
	High	1.49	9.62	2	12	11.99	0.007
Length at hatch	Control	0.31	0.13	2	30	6.87	0.032*
	Medium	0.57	0.36	2	30	22.77	3.4e − 05***
	High	0.43	0.32	2	30	14.05	0.002**
Daily growth rate	Control	0.35	0.29	2	30	0.48	0.783
	Medium	2.03	1.19	2	30	14.40	0.002***
	High	0.91	0.06	2	30	3.53	0.34

**Table 3:** Mean effect size (lnRR) ± 95% confidence interval of low (2 × 2 h air exposure day^−1^) and high intertidal (5 + 9 h air exposure day^−1^) compared to subtidal treatment (no air exposure) at three different CO_2_ rearing levels (Control = 400 µatm; Medium = 1600 µatm; High = 3000 µatm CO_2_) for different parameters in herring larvae. Significant differences in bold.

CO_2_	Intertidal	Hatch success	Size at hatch	Size at 5dph	Growth rate day^−1^	Yolksac depletion day^−1^	Cumulative mortality
Control	Low	0.07 ± 0.12	− 0.02 ± 0.05	0.00 ± 0.02	0.20 ± 0.96	0.05 ± 0.11	− 0.42 ± 0.91
Control	High	**0.13 ± 0.12**	− 0.03 ± 0.05	− **0.02 ± 0.02**	0.24 ± 0.99	0.02 ± 0.11	− **1.21 ± 0.62**
Medium	Low	0.12 ± 0.54	− **0.02 ± 0.01**	0.03 ± 0.05	0.77 ± 0.92	0.07 ± 0.19	− 0.73 ± 0.81
Medium	High	0.17 ± 0.55	− **0.06 ± 0.01**	0.03 ± 0.04	**1.10 ± 0.90**	0.08 ± 0.19	− **1.61 ± 0.82**
High	Low	− 0.04 ± 0.13	− 0.01 ± 0.02	− 0.01 ± 0.02	0.03 ± 0.56	0.03 ± 0.08	− **1.18 ± 0.85**
High	High	− 0.07 ± 0.2	− **0.05 ± 0.01**	− 0.01 ± 0.05	0.44 ± 0.59	0.03 ± 0.09	− **1.32 ± 0.82**

Overall hatch success was very high in all treatments, and ANOVA did not show a significant affect of CO_2_ (p = 0.203; F_2_ = 1.75), air exposure (p = 0.668, F_2_ = 0.41) nor the interaction (p = 0.768, F_4_ = 0.45). Effect size analysis showed a significant positive effect of tidal exposure in the ambient CO_2_ treatment, with 14% higher hatch rate in the high intertidal treatment compared to the subtidal treatment (Table 3, Fig. 4B).

Figure 4C shows the effect of air exposure and CO_2_ on larval size at hatch. Both CO_2_ and tidal exposure were significant (CO_2_: p = 0.007, *X*^2^ (2, N = 30) = 9.8; Tide: *X*^2^ (2, N = 30) = 40.6), with length negatively correlated with air exposure. The greatest effect was seen in the medium CO_2_ treatment, where short air exposure lead to 2% smaller larvae in the low intertidal treatment compared to no air exposure, and long air exposure in the high intertidal treatment lead to 6% smaller larvae compared to no air exposure in the subtidal treatment (Table 3). Length at hatch was also significantly affected by CO_2_ treatment, with smaller larvae hatching in the medium and high CO_2_ treatment with prolonged air exposure compared to control CO_2_ conditions. For larvae reared in high CO_2_ conditions, only long air exposure in the high intertidal treatment had a significant effect on size at hatch with larvae 5% smaller than those with no air exposure in the subtidal treatment (for a summary of statistics, see Tables 2 and 3).

### Carry-over effects on the larval phase

The greatest effect of air exposure was the reduction in cumulative larval mortality from hatch to yolk-sac depletion at 6 dph (Fig. 4D). Larval mortality was significantly affected by tidal exposure (p < 0.001, F_2_ = 11.21), but not CO_2_ (p = 0.198, F_2_ = 1.78) nor the interaction (p = 0.869, F_4_ = 0.31). Significant differences in mortality were found in all air exposed treatments relative to the subtidal, fully immersed treatment, with a 70%—80% reduction in larval mortality with prolonged air exposure in the high intertidal across CO_2_ treatments (Table 3).

Analyses of larval morphology, including length and height, wet and dry weight, yolk sac area and depletion rate, spinal and cranial morphology, and eye diameter revealed no significant effects of CO_2_ and air exposure, either alone or in combination with a pca. The daily growth rate computed for individual larvae from length at 5 dph relative to the mean length at hatch for the respective replicate, was significantly affected by air exposure (p = 0.002, *X*^2^ (2, N = 30) = 12.46), with growth rate increasing with increasing air exposure in the medium CO_2_ treatment (Tables 2 and 3). Interestingly, yolk sac depletion rate was not significantly affected by CO_2_ (p = 0.75, *X*^2^ (2, 30) = 0.57) or air exposure (p = 0.26, *X*^2^ (2, 30) = 2.71), and there was no significant interaction (p = 0.92, *X*^2^ (4, 30) = 0.96). Cranial abnormalities were found in very few the larvae and these were not correlated with treatment or other morphometric components in a pca.

**Figure 5:**
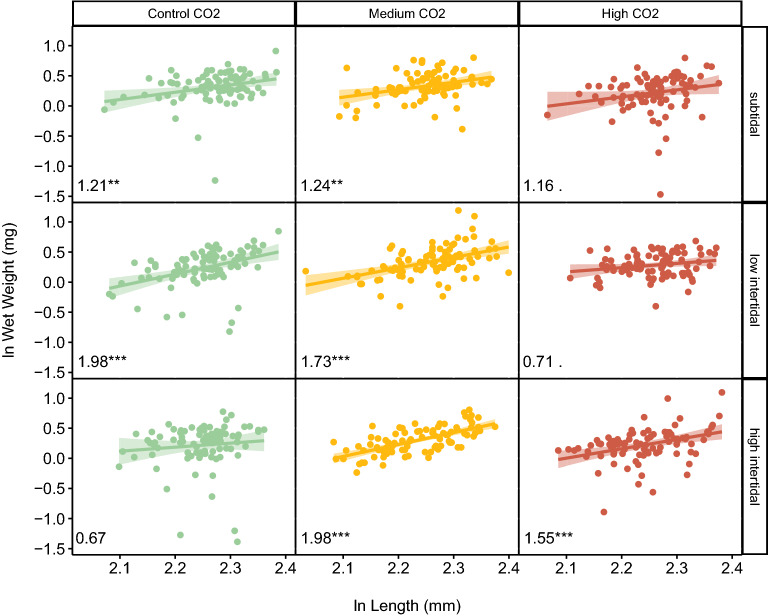
The weight to length relationship is positively impacted by air exposure with increasing CO_2_ level. Relationship between (natural logarithm of) wet weight and length of larvae for each of the CO_2_ treatments (Control = 400 µatm; Medium = 1600 µatm; High = 3000 µatm CO_2_) and tidal regimes (subtidal = no air exposure; low intertidal = 2 × 2 h air exposure day^−1^; high intertidal = 5 + 9 h air exposure day^−1^) showing all individual larvae measured with smoothed linear regression and 95% CI. Numbers at the bottom of each panel show the slope of the relationship, with the significance of the fitted regression indicated by stars (Signif. codes: 0.001 > ***; 0.01 > **; 0.05 > *; 0.1 >).

Larval dry weight: wet weight was significantly affected by CO_2_ (p = 0.003, F_2_ = 1.66) and the interaction between CO_2_ and tide was marginally significant (p = 0.060, F_4_ = 2.26). In a linear mixed model relating wet weight to length, CO_2_ and tidal exposure, length and CO_2_ were significant (length: p < 0.001, *X*^2^ (1, N = 10) = 139.3; CO_2_: p = 0.001, *X*^2^ (2, N = 10) = 13.08) and the interaction of the three parameters was marginally significant (p = 0.08, *X*^2^ (4, N = 10) = 8.12). Figure 5 shows the relationship between wet weight and length for different CO_2_ and tidal treatments. The control treatment had a higher slope with moderate air exposure in the low intertidal treatment, indicating improved condition in this treatment compared to the subtidal treatment (Fig. 5). Prolonged air exposure in the high intertidal, on the other hand, significantly increased the slope and therefore improved condition in the medium and high CO_2_ treatments. Note that the slopes of weight-length relationships in the low intertidal control treatment and the high intertidal medium CO_2_ treatment are the same, indicating that prolonged air exposure improved larval condition in the medium treatment up to the levels of the control. In contrast, in the high intertidal treatment condition was improved with air exposure but not up to levels seen in the high CO_2_ treatment (Fig. 5).

## Discussion

This study uniquely explores the role of different aquatic CO_2_ levels, in the presence and absence of air exposure, in the early life history of an important marine fish. We hypothesized that air exposure during embryonic development of Pacific herring would reduce negative effects of ocean acidification on embryonic development and hatch success, with positive carry-over effects on larval growth, development and survival. Contrary to our hypothesis and previously published studies on larval herring^15,16,18^, ocean acidification did not affect embryonic survival and development, nor cause cranial and spinal malformations in our herring larvae. Metabolic rate in embryos, implied from heart beat frequency, was elevated only in the high CO_2_ treatment with moderate air exposure, but not prolonged air exposure, showing no consistent rescue effect of air exposure on metabolic rate. Air exposure, however, did have strong beneficial effects on larval survival with the effects increasing with aquatic CO_2_ level and air exposure, implying that air exposure during embryonic development has strong carry-over effects for larval survival. Growth rates were faster in larvae where embryos had been exposed to air in the medium CO_2_ treatment, but not in the control or high CO_2_ treatment. On the other hand, larval condition, implied from weight-length ratio, decreased with high ocean acidification, but was partially restored by prolonged air exposure. Our results show that air exposure during embryonic development has strong carry-over effects on larvae and can potentially mitigate negative effects on larvae from ocean acidification.

### Effect of CO_2_ in fully immersed embryos

None of our response parameters were affected by CO_2_ levels up to 3000 µatm in our fully immersed (subtidal) treatment. Survival and hatch success were very high in all treatments, between 70 and 90%. Only the medium CO_2_ subtidal treatment showed a large variation in hatch success, however, this was likely linked to substrate, as discussed in more detail below. CO_2_ exposure did not affect growth, development or yolk sac utilization, nor was it correlated with cranial or spinal abnormalities, as has been found in both Pacific and Atlantic herring larvae in response to elevated CO_2_^15,16^. A recent study found that 1200 µatm CO_2_ in combination with higher temperature (16 °C) led to significantly increased embryonic mortality, heart rate and larger yolk sac^15^. However, at a comparable control temperature (10 °C), embryonic survival rates in that study were similar to ours, regardless of CO_2_ level. Heart rates in our study were higher than those measured by Villalobos et al.^15^ at the same developmental stage at 10 °C, and more closely matched those measured in their control CO_2_ treatment at 16 °C. It is possible that heart rates may have been elevated due to our higher water temperature under the dissecting scope (12 °C). Nevertheless, we did not see a strong signal of ocean acidification on heart beat frequency in the fully submerged treatments.

### Combined effect of air exposure and CO_2_

Counter to expectations, embryonic survival and hatch success were significantly improved by air exposure in the control treatment, but not in the elevated CO_2_ treatments. In both the medium and the high CO_2_ treatment, the intertidal treatments had no significant effect on the embryonic mortality or hatch success. A prior study had documented 8 h air exposure 2 × day^−1^ to reduce embryonic survival by nearly 20%, noting desiccation and higher temperatures incurring higher metabolic rates as likely cause^27^. In our study, metabolism (inferred from heart rate) was significantly reduced at maximum air exposure in the medium CO_2_ treatment, but elevated at moderate air exposure in the high CO_2_ treatment. This suggests that prolonged air exposure allows embryos to reduce metabolic rates when reared in moderately elevated CO_2_ levels. In the control CO_2_ group, air exposure did not affect heart rate. It must be noted, that all heart rate measurements were performed in water and the larvae hatched before the planned air-exposure heart rate measurements could be undertaken. Interestingly, heart rates were not significantly different between CO_2_ treatments in the fully submerged group, suggesting CO_2_ on its own did not cause higher metabolic rates. Another study found heart rates increased with CO_2_ but only in combination with higher temperatures^15^. A future study should compare heart rate measurements in- and out of water for a better understanding of the effects of air exposure on heart rates.

Length at hatch was significantly reduced with prolonged air exposure, regardless of CO_2_ treatment. This is similar to the findings in Jones^27^, albeit their air exposure treatment was associated with higher temperature (12 °C) instead of lower as in our study. Therefore, we can conclude that the reduction in larval length due to air exposure is robust to the effects of air temperature.

The greatest effects of CO_2_ and air exposure was on larval survival. Cumulative larval survival from hatch to yolk-sac depletion increased by 70–80% with prolonged air exposure, with the effects increasing with aquatic CO_2_ levels. This indicates strong carry-over effects from air exposure during the embryonic phase to larval development. Previous work has shown gene expression of key metabolic genes linked to lipid metabolism to be altered in larvae reared in similarly high CO_2_ waters, likely due to insufficient oxygen supply to the tissues with consequences for late larval development^36^. We hypothesize that air exposure may have enhanced conditions for oxygen uptake by embryos, lowering metabolic costs of development and improving condition and survival. The mechanisms for the carry-over effect in the present study are yet unknown but will be an interesting topic for further investigation.

We computed the weight to length relationship as a measure of condition for our herring larvae. The slope of this relationship showed that moderate, but not prolonged air exposure in the low intertidal treatment, resulted in an increased weight for a given length, implying improved condition in the control CO_2_ treatment. On the other hand, prolonged air exposure significantly improved the weight to length relationship, implying improved condition in the elevated CO_2_ treatments, but not to the levels of the control treatment. A summary of finding are presented in Fig. 1.

### Air exposure and temperature

In our study, we are unable to disentangle air exposure and temperature variation and as such can only speculate on the modulating role that the colder temperature might have played. Interestingly, developmental rate and timing of hatch were equal across all tidal and CO_2_ treatments, even though the tidal treatments experienced much lower temperature during air exposure. Jones^27^, found herring larvae to hatch a whole day earlier when exposed to 2 × 2 h air exposure at 4 °C warmer temperatures than water. In our study, larval ATUs differed by up to 14 between the subtidal (no air exposure) and high intertidal (5 + 9 h air exposure) treatments with an experienced temperature difference of up to 8 °C, without incurring differences in time to hatch. It therefore seems that the physiology of embryonic herring is rather plastic below temperatures of 10 °C, whereas temperatures above 10 °C can significantly reduce survival, likely due to increased metabolic activity and limited O_2_ supply^37,38^. While air exposure may be able to counteract O_2_ limitation and reduce metabolic costs, exposure to air above 11 °C has been found to increase mortality by 75%^31^. A future study is needed to understand the role of temperature during air exposure and aquatic acidification.

### Effect of substrate

In our study, herring eggs were collected from the wild attached to algae. These algae, in combination with cold, humid condition outdoors, prevented the eggs from desiccating when exposed to air. These algae, however, may have also had some interaction with the embryos. While care was taken to place a random mix of algal substrates (*Sargassum*, *Phyllospadix*, *Plocamium*, *Mastocarpus*, *Callophyllis*, *Rhodymenia*, *Chondracanthus*) with attached herring eggs into each treatment, both the subtidal and high intertidal in the medium CO_2_ treatment ended up with the two pieces of Turkish towel (*Chondracanthus exasperatus*), which saw poor hatch success with high mortalities shortly before hatch. This may have been due to the densely packed eggs on these flat-bladed algae, as opposed to more dispersed egg deposition on the other types of algae, although densely packed eggs are normal for herring spawn. Other than the apparent effect the Turkish towel had on hatch success, there were no observed effects of algal substrate on the herring. A study quantifying the effects of different substrates on Pacific herring found no effect on embryonic survival and hatch success^6^. Algae, being an autotrophic organism producing O_2_ from CO_2_ may have also had some effect on the carbonate system in the micro-environment surrounding the embryos. While the system was set on partial flow-through and water was circulated around the tank, we do not know how the algae might have affected CO_2_ levels directly in contact with the embryos. Therefore, it is possible that the lack of CO_2_ effect seen in our study arose from photosynthetic activity, or other substrate chemicals on embryos. Given that this is the natural state in the ocean, this scenario is more relevant than herring embryos reared on artificial substrate. As soon as the herring hatched, algae were removed, thus larval development was not directly affected by algae.

## In conclusion

Contrary to our hypothesis, CO_2_ did not increase mortality and malformations in embryonic and larval Pacific herring in our study. Furthermore, air exposure did not increase mortality or decrease developmental and metabolic rate. While we did not see strong negative effects of elevated CO_2_ levels on herring development and cranial deformities described by prior studies, air exposure during embryonic development had striking positive effects on larval growth, condition and survival in Pacific herring, with some interactive effects with CO_2_. This study underlines the importance of considering local conditions when conducting climate change experiments. Only when we place our organism of study into the context of its environment, can we begin to understand how the future climate may impact it, on an individual, population and ecosystem level.

## Data Availability

The datasets generated during and/or analysed during the current study are available from the corresponding author upon request.
